# Crystal structure of the tetra­aqua­bis­(thio­cyanato-κ*N*)cobalt(II)–caffeine–water (1/2/4) co-crystal

**DOI:** 10.1107/S2056989017008180

**Published:** 2017-06-07

**Authors:** H. El Hamdani, M. El Amane, C. Duhayon

**Affiliations:** a Equipe Métallation, Complexes Moléculaires et Applications, Université Moulay Ismail, Faculté des Sciences, BP 11201 Zitoune, 50000 Meknès, Morocco; b CNRS, LCC (Laboratoire de Chimie de Coordination), 205, route de Narbonne, F-31077 Toulouse, France; c Université de Toulouse, UPS, INPT, LCC, F-31077 Toulouse, France

**Keywords:** crystal structure, caffeine, hydrogen bonding, single-crystal X-ray diffraction analysis

## Abstract

In the structure of the title compound [systematic name: tetra­aqua­bis­(thio­cyanato-κ*N*)cobalt(II)–1,3,7-trimethyl-1,2,3,6-tetra­hydro-7*H*-purine-2,6-dione–water (1/2/4)], [Co(NCS)_2_(H_2_O)_4_]·2C_8_H_10_N_4_O_2_·4H_2_O, the cobalt(II) cation lies on an inversion centre and is coordinated in a slightly distorted octa­hedral geometry by the oxygen atoms of four water mol­ecules and two N atoms of two *trans*-arranged thio­cyanate anions. In the crystal, the complex mol­ecules inter­act with the caffeine mol­ecules through O—H⋯N, O—H⋯O and C—H⋯S hydrogen bonds and π–π inter­actions [centroid-to-centroid distance = 3.4715 (5) Å], forming layers parallel to the *ab* plane, which are further connected into a three-dimensional network by O—H⋯O and O—H⋯S hydrogen bonds involving the non-coordinating water mol­ecules.

## Abstract 2

In the structure of the title compound, [Co(NCS)_2_(H_2_O)_4_]·2C_8_H_10_N_4_O_2_·4H_2_O, the cobalt metal lies on an inversion centre and is coordinated in a slightly distorted octa­hedral geometry. In the crystal, the complex mol­ecules inter­act with the caffeine mol­ecules through O—H⋯N, O—H⋯O, C–H⋯S hydrogen bonds and π–π inter­actions.

## Chemical context   

Compounds with supra­molecular metal–organic structures, which are diversified by their innovative applications, attract attention in various fields such as non-linear optical activity, catalysis, electrical conductivity, and cooperative magnetic behavior (Fan *et al.*, 2016 ▸). In particular, the supra­molecular complexes of mixed metals and ligands that possess active pharmaceutical ingredients (APIs) offers an approach to generate crystalline materials that form pharmaceutical co-crystals to effect therapeutic parameters such as solubility and lipophilicity (Ma & Moulton, 2007 ▸). The properties of caffeine as a pharmaceutical compound exhibiting moisture instability with the formation of a non-stoichiometric crystalline hydrate have been widely studied. Caffeine is a stimulant of the central nervous system and a smooth muscle relaxant, and is used as a formulation additive to analgesic remedies (Trask *et al.*, 2005 ▸). Caffeine has attractive effects on various biological systems, including cardiovascular, gastrointestinal, respiratory and muscle systems (Taşdemir *et al.*, 2016 ▸), and forms complexes with transition metals having different coordination and biological properties such as anti-inflammatory and anti­bacterial (Taşdemir *et al.*, 2016 ▸). Thio­cyanate is a commonly used ligand because of its numerous bonding modes to one or more transition metal ions, and provides useful precursors for numerous coordination complexes. Usually, the thio­cyanate anion bonds terminally through the nitro­gen atom with first-row transition metals, and can act as a hydrogen-bond acceptor through the nitro­gen or sulfur atom (Bie *et al.*, 2005 ▸).
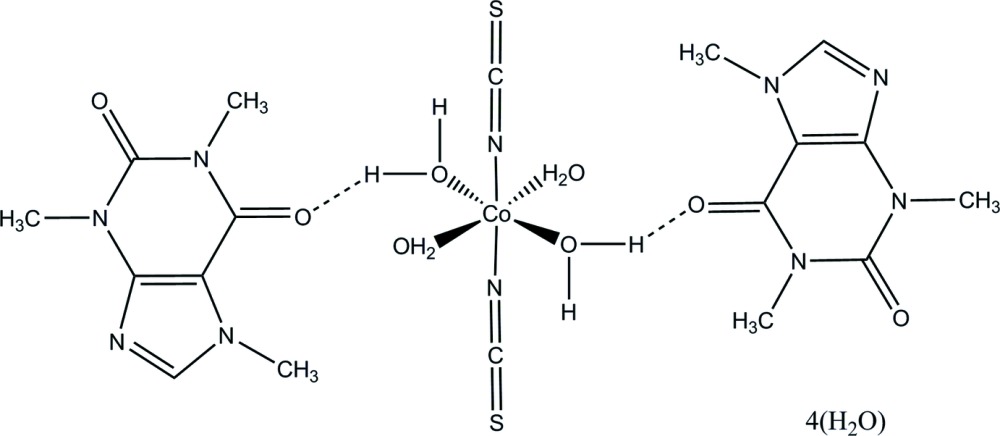


## Structural commentary   

**Figure 1:**
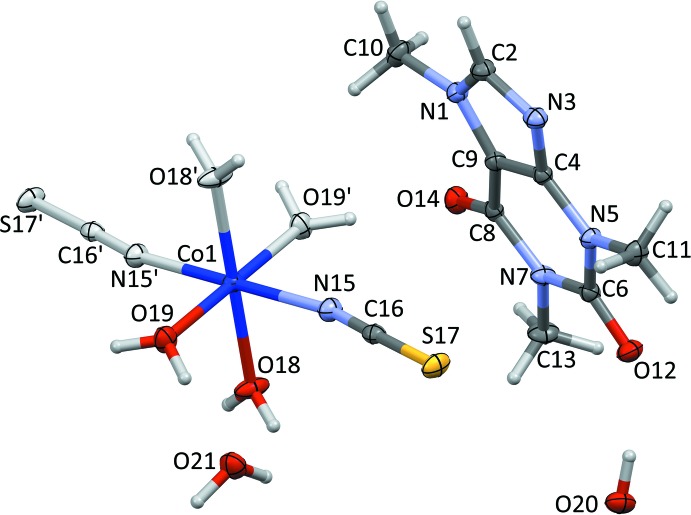
The asymmetric unit [expanded for the cobalt(II) cation to show the full coordination sphere; primed atoms are related to the non-primed atoms by the symmetry operation −*x* + 2, −*y* + 1, −*z* + 1] of the title compound, with displacement ellipsoids drawn at the 50% probability level

The asymmetric unit of the title compound (Fig. 1 ▸) contains half a complex mol­ecule of formula [Co(NCS)_2_(H_2_O)_4_], a caffeine mol­ecule and two free water mol­ecules. The cobalt(II) cation lies on an inversion centre and displays a *trans*-arranged octa­hedral coordination geometry provided by the N atoms of two thio­cyanate anions and four O atoms of coordinating water mol­ecules. The Co1—N15 [2.0981 (8) Å] and Co1—O18 [2.0981 (7) Å] bond lengths are equal within standard uncertainties and significantly longer than the Co1–O19 bond length [2.0732 (7) Å], and therefore the CoN_2_O_4_ octa­hedron is slightly axially compressed. This structural feature is typical for related compounds (Shylin *et al.*, 2013 ▸, 2015 ▸). The thio­cyanato ligands are bound through the nitro­gen atoms and are nearly linear [N15—C16—S17 = 177.81 (8)°], while the Co–NCS linkage is bent [C16—N15—Co1 = 167.35 (8)°]. Previously reported complexes with an N-bound NCS group possess similar structural features (Petrusenko *et al.*, 1997 ▸). The caffeine mol­ecule is nearly planar (r.m.s. deviation = 0.0346 Å), with a maximum deviation from the mean plane of 0.0404 (7) Å for atom N5.

## Supra­molecular features   

**Figure 2:**
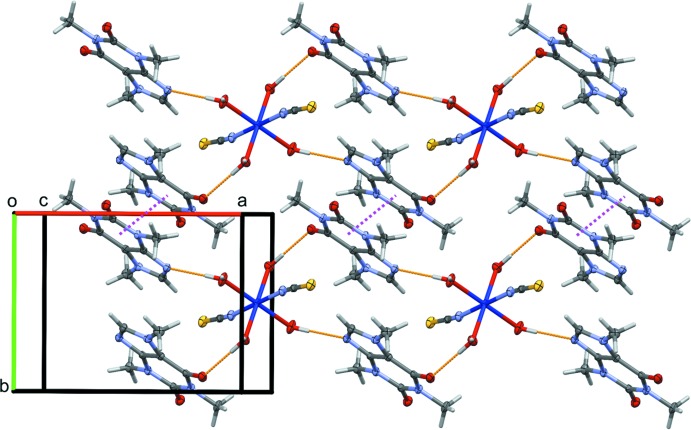
Partial packing diagram of the title compound, showing the network of hydrogen bonds (orange dotted lines) and π–π inter­actions (purple dotted lines) linking complexes and caffeine mol­ecules into layers parallel to the *ab* plane.

**Figure 3:**
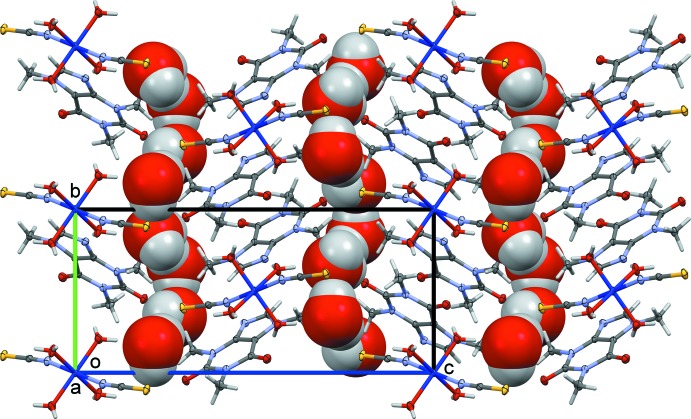
Crystal packing of the title compound viewed down the *a* axis.

In the crystal, each complex mol­ecule inter­acts with four neighboring caffeine mol­ecules through classical O—H⋯N and O—H⋯O hydrogen bonds (Table 1 ▸) involving the coordinating water mol­ecules as H-atom donors to form layers parallel to the *ab* plane. These planes are further enforced by C—H⋯S hydrogen bonds and π–π inter­actions occurring between centrosymmetrically related six-membered rings of the purine ring system [*Cg*⋯*Cg* ^i^ = 3.4715 (5) Å; *Cg* is the centroid of the N3/N7/C4/C6/C8/C9 ring; symmetry code: (i) 1 − *x*, 2 − *y*, 1 − *z*; Fig. 2 ▸], and are alternated by layers of non-coordinating water mol­ecules linked through O—H⋯O and O—H⋯S hydrogen bonds (Fig. 3 ▸), leading to the formation of a three-dimensional network (Fig. 3 ▸).

## Synthesis and crystallization   

In a glass tube, a solution of CoCl_2_·6H_2_O (129 mg, 1 mmol) in 5 ml of water and caffeine (194.19 mg, 1 mmol) in 10 ml of ethanol was added to a solution of potassium thio­cyanate (190 mg, 2 mmol) in 5 ml of water. Single crystals of the title compound suitable for X-ray analysis were grown after several months by slow evaporation of the solvent at room temperature.

## Refinement   

Crystal data, data collection and structure refinement details are summarized in Table 2 ▸. All H atoms could be located in a difference-Fourier map, but those attached to carbon atoms were repositioned geometrically. The H atoms were initially refined with soft restraints on the bond lengths and angles to regularize their geometry (C—H = 0.98, O—H = 0.82 Å) and with *U*_iso_(H) set at 1.2–1.5 times of the *U*_eq_ of the parent atom, after which the positions were refined with riding constraints (Cooper *et al.*, 2010 ▸).

## Supplementary Material

Crystal structure: contains datablock(s) global, I. DOI: 10.1107/S2056989017008180/rz5216sup1.cif

Structure factors: contains datablock(s) I. DOI: 10.1107/S2056989017008180/rz5216Isup2.hkl

CCDC reference: 1553654

Additional supporting information:  crystallographic information; 3D view; checkCIF report

## Figures and Tables

**Table 1:** Hydrogen-bond geometry (Å, °)

*D*—H⋯*A*	*D*—H	H⋯*A*	*D*⋯*A*	*D*—H⋯*A*
C2—H21⋯S17^i^	0.97	2.83	3.7622 (9)	160.6
O20—H202⋯O21^ii^	0.86	1.98	2.8119 (11)	161.6
O19—H191⋯O21	0.86	1.91	2.7634 (10)	174.9
O18—H182⋯N3^iii^	0.85	2.01	2.8671 (11)	178.4
O21—H211⋯S17^iv^	0.88	2.38	3.2481 (7)	173.3
O21—H212⋯O20^iv^	0.87	1.97	2.8157 (11)	164.8
O20—H201⋯O12	0.85	2.02	2.8531 (10)	166.8
O19—H192⋯O14^v^	0.85	1.89	2.7460 (10)	178.5

Symmetry codes: (i) 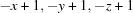; (ii) 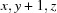; (iii) 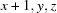; (iv) 
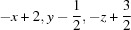
; (v) 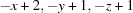.

**Table 2:** Experimental details

Crystal data	
Chemical formula	[Co(NCS)_2_(H_2_O)_4_]·2C_8_H_10_N_4_O_2_·4H_2_O
*M*_r_	707.61
Crystal system, space group	Monoclinic, *P*2_1_/*c*
Temperature (K)	120
*a*, *b*, *c* (Å)	10.65854 (19), 8.16642 (14), 18.0595 (3)
β (°)	96.4701 (15)
*V* (Å^3^)	1561.93 (3)
*Z*	2
Radiation type	Mo *K*α
μ (mm^−1^)	0.75
Crystal size (mm)	0.25 × 0.20 × 0.20

Data collection	
Diffractometer	Oxford Diffraction Gemini
Absorption correction	Multi-scan (*CrysAlis PRO*; Agilent, 2011 ▸)
*T*_min_, *T*_max_	0.78, 0.86
No. of measured, independent and observed [*I* > 2.0σ(*I*)] reflections	62568, 4002, 3693
*R*_int_	0.023
(sin θ/λ)_max_ (Å^−1^)	0.689

Refinement	
*R*[*F* ^2^ > 2σ(*F* ^2^)], *wR*(*F* ^2^), *S*	0.023, 0.022, 1.13
No. of reflections	3586
No. of parameters	196
H-atom treatment	H-atom parameters not refined
Δρ_max_, Δρ_min_ (e Å^−3^)	0.36, −0.24

Computer programs: *GEMINI* (Oxford Diffraction, 2006 ▸), *CrysAlis PRO* (Agilent, 2011 ▸), *SIR92* (Altomare *et al.*, 1994 ▸), *CRYSTALS* (Betteridge *et al.*, 2003 ▸) and *CAMERON* (Watkin *et al.*, 1996 ▸). Weighting scheme: Chebychev polynomial, (Watkin, 1994 ▸; Prince, 1982 ▸).

## supplementary crystallographic information

### Crystal data

**Table:**

[Co(NCS)_2_(H_2_O)_4_]·2C_8_H_10_N_4_O_2_·4H_2_O	*F*(000) = 738
*M**_r_* = 707.61	*D*_x_ = 1.504 Mg m^−^^3^
Monoclinic, *P*2_1_/*c*	Mo *K*α radiation, λ = 0.71073 Å
Hall symbol: -P 2ybc	Cell parameters from 26895 reflections
*a* = 10.65854 (19) Å	θ = 4–29°
*b* = 8.16642 (14) Å	µ = 0.75 mm^−^^1^
*c* = 18.0595 (3) Å	*T* = 120 K
β = 96.4701 (15)°	Block, orange
*V* = 1561.93 (3) Å^3^	0.25 × 0.20 × 0.20 mm
*Z* = 2	

### Data collection

**Table:**

Oxford Diffraction Gemini diffractometer	3693 reflections with *I* > 2.0σ(*I*)
Graphite monochromator	*R*_int_ = 0.023
φ & ω scans	θ_max_ = 29.3°, θ_min_ = 3.1°
Absorption correction: multi-scan (CrysAlis PRO; Agilent, 2011)	*h* = −14→13
*T*_min_ = 0.78, *T*_max_ = 0.86	*k* = −10→10
62568 measured reflections	*l* = −24→24
4002 independent reflections	

### Refinement

**Table:**

Refinement on *F*	Primary atom site location: structure-invariant direct methods
Least-squares matrix: full	Hydrogen site location: difference Fourier map
*R*[*F*^2^ > 2σ(*F*^2^)] = 0.023	H-atom parameters not refined
*wR*(*F*^2^) = 0.022	Method, part 1, Chebychev polynomial, (Watkin, 1994, *P*rince, 1982) [*w*eight] = 1.0/[A_0_*T_0_(x) + A_1_*T_1_(x) ··· + A_n-1_]*T_n-1_(x)] where A_i_ are the Chebychev coefficients listed belo*w* and x = *F* /*F*max Method = Robust Weighting (*P*rince, 1982) W = [*w*eight] * [1-(delta*F*/6*sigma*F*)^2^]^2^ A_i_ are: 4.58 -1.83 2.76
*S* = 1.13	(Δ/σ)_max_ = 0.001
3586 reflections	Δρ_max_ = 0.36 e Å^−^^3^
196 parameters	Δρ_min_ = −0.24 e Å^−^^3^
0 restraints	

### Special details

**Table:**

Experimental. The crystal was placed in the cold stream of an Oxford Cryosystems open-flow nitrogen cryostat (Cosier & Glazer, 1986) with a nominal stability of 0.1K. Cosier, J. & Glazer, A.M., 1986. J. Appl. Cryst. 105-107.

### Fractional atomic coordinates and isotropic or equivalent isotropic displacement parameters (Å^2^)

**Table:**

	*x*	*y*	*z*	*U*_iso_*/*U*_eq_	
N1	0.52963 (7)	0.70159 (10)	0.44118 (4)	0.0159	
N3	0.39238 (7)	0.69668 (10)	0.52732 (4)	0.0165	
N5	0.50891 (7)	0.88202 (9)	0.61607 (4)	0.0138	
N7	0.70166 (7)	0.97981 (10)	0.57845 (4)	0.0148	
N15	0.87722 (7)	0.56972 (10)	0.57738 (4)	0.0177	
C2	0.42006 (9)	0.64292 (12)	0.46092 (5)	0.0177	
C4	0.49202 (8)	0.79469 (11)	0.55076 (5)	0.0134	
C6	0.61761 (8)	0.97269 (11)	0.63265 (5)	0.0145	
C8	0.68939 (8)	0.89801 (11)	0.50972 (5)	0.0140	
C9	0.57826 (8)	0.80031 (11)	0.49965 (5)	0.0138	
C10	0.58693 (10)	0.66144 (13)	0.37365 (5)	0.0214	
C11	0.41902 (9)	0.86319 (12)	0.67132 (5)	0.0187	
C13	0.81636 (9)	1.07902 (13)	0.59652 (6)	0.0212	
C16	0.82097 (8)	0.58619 (11)	0.62832 (5)	0.0146	
O12	0.63887 (6)	1.04807 (9)	0.69164 (4)	0.0199	
O14	0.76700 (6)	0.91542 (9)	0.46473 (4)	0.0191	
O18	1.14207 (7)	0.63274 (10)	0.56379 (4)	0.0243	
O19	1.04404 (7)	0.29467 (9)	0.56531 (4)	0.0202	
O20	0.85940 (7)	1.15725 (10)	0.78244 (4)	0.0218	
O21	1.04346 (7)	0.33657 (9)	0.71711 (4)	0.0227	
S17	0.73704 (2)	0.60472 (3)	0.699098 (13)	0.0206	
Co1	1.0000	0.5000	0.5000	0.0133	
H21	0.3674	0.5683	0.4293	0.0226*	
H103	0.6697	0.6105	0.3874	0.0339*	
H102	0.5958	0.7614	0.3446	0.0343*	
H101	0.5302	0.5835	0.3448	0.0347*	
H111	0.4397	0.9420	0.7112	0.0299*	
H112	0.3339	0.8828	0.6471	0.0298*	
H113	0.4258	0.7533	0.6921	0.0310*	
H131	0.8546	1.0964	0.5513	0.0326*	
H132	0.7942	1.1847	0.6172	0.0322*	
H133	0.8741	1.0211	0.6328	0.0327*	
H181	1.1398	0.6463	0.6098	0.0378*	
H202	0.9034	1.2295	0.7618	0.0364*	
H191	1.0428	0.3014	0.6127	0.0336*	
H182	1.2164	0.6538	0.5531	0.0384*	
H211	1.1073	0.2809	0.7394	0.0376*	
H212	1.0591	0.4409	0.7176	0.0382*	
H201	0.7920	1.1412	0.7538	0.0355*	
H192	1.1020	0.2292	0.5552	0.0330*	

### Atomic displacement parameters (Å^2^)

**Table:**

	*U*^11^	*U*^22^	*U*^33^	*U*^12^	*U*^13^	*U*^23^
N1	0.0180 (4)	0.0155 (4)	0.0136 (3)	0.0004 (3)	0.0001 (3)	−0.0018 (3)
N3	0.0150 (3)	0.0163 (4)	0.0178 (4)	−0.0018 (3)	0.0000 (3)	−0.0004 (3)
N5	0.0130 (3)	0.0168 (4)	0.0119 (3)	−0.0009 (3)	0.0028 (3)	−0.0008 (3)
N7	0.0127 (3)	0.0175 (4)	0.0140 (3)	−0.0031 (3)	0.0010 (3)	0.0002 (3)
N15	0.0152 (3)	0.0229 (4)	0.0155 (3)	−0.0010 (3)	0.0039 (3)	−0.0018 (3)
C2	0.0164 (4)	0.0178 (4)	0.0185 (4)	−0.0015 (3)	−0.0005 (3)	−0.0012 (3)
C4	0.0133 (4)	0.0136 (4)	0.0132 (4)	0.0012 (3)	0.0007 (3)	0.0012 (3)
C6	0.0140 (4)	0.0156 (4)	0.0135 (4)	0.0007 (3)	0.0002 (3)	0.0007 (3)
C8	0.0137 (4)	0.0141 (4)	0.0140 (4)	0.0024 (3)	0.0012 (3)	0.0023 (3)
C9	0.0149 (4)	0.0146 (4)	0.0120 (4)	0.0011 (3)	0.0011 (3)	0.0000 (3)
C10	0.0269 (5)	0.0228 (5)	0.0152 (4)	0.0007 (4)	0.0058 (4)	−0.0028 (4)
C11	0.0177 (4)	0.0244 (5)	0.0152 (4)	−0.0010 (4)	0.0067 (3)	−0.0006 (3)
C13	0.0154 (4)	0.0269 (5)	0.0208 (4)	−0.0084 (4)	0.0000 (3)	−0.0009 (4)
C16	0.0139 (4)	0.0147 (4)	0.0146 (4)	−0.0021 (3)	−0.0005 (3)	−0.0003 (3)
O12	0.0199 (3)	0.0232 (3)	0.0163 (3)	−0.0028 (3)	0.0006 (2)	−0.0052 (3)
O14	0.0172 (3)	0.0224 (3)	0.0190 (3)	0.0006 (3)	0.0071 (2)	0.0021 (3)
O18	0.0175 (3)	0.0413 (4)	0.0147 (3)	−0.0121 (3)	0.0044 (2)	−0.0074 (3)
O19	0.0212 (3)	0.0232 (3)	0.0173 (3)	0.0041 (3)	0.0063 (2)	0.0018 (3)
O20	0.0203 (3)	0.0300 (4)	0.0146 (3)	−0.0021 (3)	−0.0004 (2)	0.0011 (3)
O21	0.0241 (3)	0.0246 (4)	0.0195 (3)	0.0044 (3)	0.0020 (3)	0.0047 (3)
S17	0.01944 (11)	0.02902 (12)	0.01456 (10)	−0.00462 (9)	0.00757 (8)	−0.00446 (9)
Co1	0.01068 (8)	0.01859 (9)	0.01093 (8)	−0.00186 (6)	0.00259 (5)	−0.00104 (6)

### Geometric parameters (Å, º)

**Table:**

N1—C2	1.3469 (12)	C10—H102	0.980
N1—C9	1.3820 (11)	C10—H101	0.985
N1—C10	1.4616 (12)	C11—H111	0.972
N3—C2	1.3407 (12)	C11—H112	0.975
N3—C4	1.3588 (12)	C11—H113	0.972
N5—C4	1.3727 (11)	C13—H131	0.963
N5—C6	1.3792 (11)	C13—H132	0.980
N5—C11	1.4672 (11)	C13—H133	0.969
N7—C6	1.4006 (11)	C16—S17	1.6476 (9)
N7—C8	1.4027 (11)	O18—Co1	2.0981 (7)
N7—C13	1.4723 (11)	O18—H181	0.842
N15—C16	1.1610 (12)	O18—H182	0.853
N15—Co1	2.0981 (8)	O19—Co1	2.0732 (7)
C2—H21	0.969	O19—H191	0.860
C4—C9	1.3749 (12)	O19—H192	0.853
C6—O12	1.2291 (11)	O20—H202	0.864
C8—C9	1.4226 (12)	O20—H201	0.846
C8—O14	1.2314 (11)	O21—H211	0.877
C10—H103	0.982	O21—H212	0.868
			
C2—N1—C9	105.49 (7)	H111—C11—H112	110.1
C2—N1—C10	126.68 (8)	N5—C11—H113	109.5
C9—N1—C10	127.76 (8)	H111—C11—H113	109.0
C2—N3—C4	103.21 (8)	H112—C11—H113	110.5
C4—N5—C6	119.42 (7)	N7—C13—H131	108.3
C4—N5—C11	119.83 (7)	N7—C13—H132	109.8
C6—N5—C11	120.37 (7)	H131—C13—H132	109.6
C6—N7—C8	126.55 (7)	N7—C13—H133	109.2
C6—N7—C13	116.65 (7)	H131—C13—H133	110.3
C8—N7—C13	116.77 (7)	H132—C13—H133	109.6
C16—N15—Co1	167.35 (8)	N15—C16—S17	177.81 (8)
N1—C2—N3	113.89 (8)	Co1—O18—H181	120.7
N1—C2—H21	122.0	Co1—O18—H182	127.7
N3—C2—H21	124.1	H181—O18—H182	109.1
N5—C4—N3	126.58 (8)	Co1—O19—H191	119.2
N5—C4—C9	121.78 (8)	Co1—O19—H192	120.6
N3—C4—C9	111.64 (8)	H191—O19—H192	110.2
N7—C6—N5	117.28 (8)	H202—O20—H201	107.9
N7—C6—O12	120.99 (8)	H211—O21—H212	111.4
N5—C6—O12	121.70 (8)	O18^i^—Co1—O18	179.995
N7—C8—C9	111.93 (7)	O18^i^—Co1—N15^i^	87.69 (3)
N7—C8—O14	121.76 (8)	O18—Co1—N15^i^	92.31 (3)
C9—C8—O14	126.29 (8)	O18^i^—Co1—N15	92.31 (3)
C8—C9—N1	131.30 (8)	O18—Co1—N15	87.69 (3)
C8—C9—C4	122.88 (8)	N15^i^—Co1—N15	179.995
N1—C9—C4	105.77 (8)	O18^i^—Co1—O19	89.86 (3)
N1—C10—H103	109.4	O18—Co1—O19	90.14 (3)
N1—C10—H102	109.5	N15^i^—Co1—O19	92.36 (3)
H103—C10—H102	110.5	N15—Co1—O19	87.64 (3)
N1—C10—H101	107.3	O18^i^—Co1—O19^i^	90.14 (3)
H103—C10—H101	109.9	O18—Co1—O19^i^	89.86 (3)
H102—C10—H101	110.2	N15^i^—Co1—O19^i^	87.64 (3)
N5—C11—H111	108.8	N15—Co1—O19^i^	92.36 (3)
N5—C11—H112	108.9	O19—Co1—O19^i^	179.994

Symmetry code: (i) −*x*+2, −*y*+1, −*z*+1.

### Hydrogen-bond geometry (Å, º)

**Table:**

*D*—H···*A*	*D*—H	H···*A*	*D*···*A*	*D*—H···*A*
C2—H21···S17^ii^	0.97	2.83	3.7622 (9)	160.6
O20—H202···O21^iii^	0.86	1.98	2.8119 (11)	161.6
O19—H191···O21	0.86	1.91	2.7634 (10)	174.9
O18—H182···N3^iv^	0.85	2.01	2.8671 (11)	178.4
O21—H211···S17^v^	0.88	2.38	3.2481 (7)	173.3
O21—H212···O20^v^	0.87	1.97	2.8157 (11)	164.8
O20—H201···O12	0.85	2.02	2.8531 (10)	166.8
O19—H192···O14^i^	0.85	1.89	2.7460 (10)	178.5

Symmetry codes: (i) −*x*+2, −*y*+1, −*z*+1; (ii) −*x*+1, −*y*+1, −*z*+1; (iii) *x*, *y*+1, *z*; (iv) *x*+1, *y*, *z*; (v) −*x*+2, *y*−1/2, −*z*+3/2.
